# Mass Spectrometry Based Proteomic Analysis of Salivary Glands of Urban Malaria Vector *Anopheles stephensi*

**DOI:** 10.1155/2014/686319

**Published:** 2014-07-14

**Authors:** Sonam Vijay, Manmeet Rawat, Arun Sharma

**Affiliations:** ^1^Protein Biochemistry and Structural Biology Laboratory, National Institute of Malaria Research (ICMR), Sector-8, Dwarka, New Delhi 110077, India; ^2^Department of Internal Medicine, University of New Mexico School of Medicine, Albuquerque, NM 87106, USA

## Abstract

Salivary gland proteins of *Anopheles* mosquitoes offer attractive targets to understand interactions with sporozoites, blood feeding behavior, homeostasis, and immunological evaluation of malaria vectors and parasite interactions. To date limited studies have been carried out to elucidate salivary proteins of *An. stephensi* salivary glands. The aim of the present study was to provide detailed analytical attributives of functional salivary gland proteins of urban malaria vector *An. stephensi*. A proteomic approach combining one-dimensional electrophoresis (1DE), ion trap liquid chromatography mass spectrometry (LC/MS/MS), and computational bioinformatic analysis was adopted to provide the first direct insight into identification and functional characterization of known salivary proteins and novel salivary proteins of *An. stephensi*. Computational studies by online servers, namely, MASCOT and OMSSA algorithms, identified a total of 36 known salivary proteins and 123 novel proteins analysed by LC/MS/MS. This first report describes a baseline proteomic catalogue of 159 salivary proteins belonging to various categories of signal transduction, regulation of blood coagulation cascade, and various immune and energy pathways of *An. stephensi* sialotranscriptome by mass spectrometry. Our results may serve as basis to provide a putative functional role of proteins in concept of blood feeding, biting behavior, and other aspects of vector-parasite host interactions for parasite development in anopheline mosquitoes.

## 1. Background

Malaria has been prevalent for a long time in tropical developing regions causing great morbidity and mortality [1]. The world malaria report 2013 [1] released by the World Health Organization (WHO) states that an estimated 3.4 billion people are at risk of malaria and around 207 million cases of malaria occurred globally. Among the malaria vectors,* An. stephensi* is an important urban malaria vector of Indo-Pakistan subcontinent [2]. Due to susceptible nature of* An. stephensi* to both human and rodent malaria species, it turns out to be significant to use as a reference laboratory model to study salivary gland-parasite interactions [3]. Salivary glands of mosquitoes perform various functions for survival of the vectors and also are conducive for blood feeding, harbouring of malaria parasites, and eventual parasite transmission. Salivary secretions have various pharmacological substances such as inhibitors of the clotting cascade, inhibitors of vasoconstricting substances, and inhibitors of platelet aggregation, which are necessary for continuous blood feeding in mosquitoes [4].

The salivary gland proteins are thus relevant for malaria research since the* Plasmodium* sporozoites invade the salivary glands and are injected with the saliva into vertebrate hosts during blood feeding [5]. In addition to this, various other functions are also performed by salivary glands as sugar feeding [6] and blood feeding [4], and some salivary gland proteins show immunogenic properties [7] that help in modulating the immune response of the human host and salivary proteins were found to be annotated in insecticide resistant* Culex* mosquitoes [8].

Salivary gland tissues of* An. stephensi* have been studied for molecular and genetic studies and for malaria transmission. Many products of salivary gland gene expression have been studied in* An. stephensi* with help of applications of transcriptomics and proteomics [3]. However, proteomics studies have also been described and roles of some putative salivary proteins were also proposed in evolution of blood feeding and in the discovery of novel antihemostatic substances [3]. However, such* An. stephensi* sialome studies were elucidated using transcriptomic studies that include full-length cDNA library sequence of* An. stephensi* [3, 5] and during our EST studies on* An. stephensi* salivary glands [9, 10]. Though proteomic studies along with transcriptomics studies have been carried out in* An. gambiae* salivary gland [3] with large number of diverse predicted salivary proteins [11], thus far no comprehensive and detailed functional properties of salivary gland proteins of* Anopheles stephensi* have been studied. Hence, to fully understand high biological actions of salivary gland proteins and to elucidate their role in different biosynthetic pathways, application of proteomics is very much needed. Mass spectrometry based proteomics data, when applied in conjunction with mosquito salivary gland genomic and transcriptomics databases, provides a comprehensive account that can be used to identify proteins as putative functional components of the salivary glands for novel malaria control strategies [3].

Unfortunately, to date, only limited studies exist to efficiently explore molecular interactions and role of salivary gland proteins of the mosquito and the sporozoites of the* Plasmodium* parasite. Transcriptomics studies combined with genetic variations across evolutionarily related mosquitoes for targeting specific RNA sequences are generally inconsistent to generate functional proteomic data sets [3, 9, 10]. Gel electrophoresis (1DE) along with mass spectrometry and detailed bioinformatic analysis is a powerful and direct tool to study global protein profiling in tissues. Therefore, as a first step, the aim of the present study was to identify and characterize the protein profiles of* An. stephensi* salivary gland in order to establish functional phylogeny among different anophelines and other mosquitoes to validate their evolutionary functions.

Here we describe an in-gel proteomic approach using 1D and LC-MS/MS to characterize the proteome of the salivary gland extracts (SGEs) of* An. stephensi.* We have achieved this by analyzing mass spectrometry data using MASCOT and OMSSA algorithm. We report, herewith, the catalogue of 159 known and novel proteins obtained from LC-MS/MS data through a detailed bioinformatics analysis which should serve as a first preliminary step for putative functional identification of several salivary glands extracts (SGEs) proteins and proteomes at molecular levels that may provide novel targets for interrupting parasitic transmission life cycle. Our study thus opens up the possibilities of elucidating salivary gland-parasite interactions during blood meals and may provide relevant baseline information for characterizing proteomes of other mosquitoes for development of novel vector control strategies.

## 2. Materials and Methods

### 2.1. Sample Preparation

#### 2.1.1. Mosquitoes

*Anopheles stephensi* mosquitoes reared in our insectary (National Institute of Malaria Research, India) were used in this study. 3-4-day-old sugar fed mosquitoes were used in the experiments and were maintained and reared under identical standard conditions at 27°C ± 2°C with 70% ± 10% relative humidity and a photoperiod of 12 : 12 (light/dark) hours. Adult mosquitoes were maintained on a 10% sucrose solution.

#### 2.1.2. Dissection of Salivary Glands

*Anopheles stephensi* salivary glands were dissected on slide using fine needles under a stereomicroscope at 4x magnification using phosphate-buffered saline (PBS) and were pooled. After dissection the tissues were immediately placed in a PBS buffer (100 *μ*L) with protease inhibitors (Complete, Roche Diagnostics, Germany) and stored at −80°C until use.

#### 2.1.3. Salivary Glands Extract Preparation

A total of 100 pairs of salivary glands of female* An. stephensi* were used to prepare salivary gland extracts (SGEs). Dissected salivary glands (100 pairs) in PBS were sonicated on ice with three pulses for 20 sec. Afterward the suspension was centrifuged for 10 min at 5000 rpm at 4°C to remove cell debris. The extracted supernatant was collected and stored at −80°C for further analysis. Protein estimation was carried out by Lowry's method [12] and analyzed by bovine serum albumin BSA standard curve. The SGEs were stored for in-gel trypsin digestion for further analysis.

### 2.2. Sample Analysis

#### 2.2.1. 1D Gel Electrophoresis (SDS-PAGE)

SGE samples were first fractionated on SDS-PAGE for separation. Briefly, 50–75 *μ*g of SGE sample was dissolved in sample buffer (0.625 M Tris HCl, 10% SDS, glycerol, and distilled water) containing *β*-mercaptoethanol (10% vol/vol) and heated at 95°C for 5 min. 30 *μ*L sample was then loaded onto an acrylamide gel (3% stacking gel and 10% resolving gel) and subjected to electrophoresis on a Bio-Rad apparatus (Bio-Rad, USA). Protein molecular weight markers (Genei protein range marker, Bangalore Genei) were also run on the gel. The gel was silver-stained according to the manufacturer's protocol (G-Biosciences). Stained gel was then sliced into different bands and these gel bands were individually subjected to digestion with proteomic grade trypsin (Roche Diagnostics, USA).

#### 2.2.2. In-Gel Protein Digestion before Identification by LC/MS/MS

Proteins were reduced, alkylated with iodoacetamide, and digested with trypsin overnight at 37°C. Briefly, the excised gel slices were subjected to reduction and were dried in a vacuum centrifuge. DTT (10 mM) in ammonium bicarbonate (100 mM) was added to gel pieces and proteins were reduced for 1 hour at 56°C. After cooling to room temperature reduced proteins were alkylated with IAA (55 mM) in ammonium bicarbonate (100 mM) for 45 min at 25°C. After incubation in the dark with occasional vortexing the gel pieces were washed with 50–100 *μ*L of ammonium bicarbonate (100 mM) for 10 min, dehydrated by addition of acetonitrile, swelled by rehydration in ammonium bicarbonate (100 mM), and shrunk again by addition of the same volume of acetonitrile. After removal of the liquid phase, the gel pieces were completely dried in a vacuum centrifuge. Gel slices were then swollen in a digestion buffer containing ammonium bicarbonate (50 mM), CaCl_2_ (5 mM), and trypsin solution (12.5 ng/*μ*L) (ratio 1 : 100) in an ice cold bath. The supernatant was removed after 45 mins and replaced with 5–10 *μ*L of the same buffer, but without trypsin, to keep the gel pieces wet during enzymatic cleavage (37°C, overnight). Peptides were extracted by one change of ammonium bicarbonate (20 mM) and three changes of 5% formic acid in acetonitrile (50%) (20 min for each change) at room temperature and dried down. This peptide mixture was then stored for analysis by LC/MS/MS.

### 2.3. Instrumentation and Analysis

#### 2.3.1. LC/MS/MS

In-gel digested peptides were analyzed by nano LC-ESI-QTOF-MS/MS on a Bruker micrOTOF-Q II system. For LC-MS/MS analysis 15 *μ*L of each sample were injected. Peptides were first trapped and preconcentrated on a C-18 precolumn (Dionex) at 30 *μ*L flow for 5 min and later eluted on the separation column with a flow of 220 nL/min (column dimensions were I.D. 75 *μ*m, length 15 cm, PepMap C-18, 3 *μ*m, 100 Å). Solvents used for elution of peptides were solvent A: water, ACN (1.0%), and formic acid (0.1%) and solvent B: ACN and formic acid (0.1%). All samples were measured in “auto” MS/MS mode, positive ion mode on the Bruker nanospray source with a capillary voltage 1,500 Volts, dry gas flow 6.0 L/min, dry temperature: 130°C, 1 MS followed by 5 MS/MS of the most intense ions, total cycle time 4.4–8.8 sec,* m*/*z* 400–1,400 taken as precursor ions for MS/MS, active exclusion after 2 spectra for 0.5 min, and threshold for MS/MS set to 1,000.

### 2.4. Database Search

*MASCOT Server*. LC/MS/MS data were analyzed using Bruker Daltonics ProteinScape database system. Raw data were converted into MGF format and database searches were performed on a MASCOT server (MASCOT 2.2, MS/MS Ion Search) using fixed modification (none), variable modification carbamidomethyl (Cys), and methionine oxidation for each protein band sample (16 digested samples). Parameters used were trypsin as an enzyme, max missed cleavages: 1, peptide mass tolerance: ±0.05 Da or 10 ppm, fragment mass tolerance: ±0.05 Da, data format: MASCOT generic, instrument type: ESI-QUAD-TOF, and databases used SwissProt and NCBInr. Searches were made against* Anopheles* and other mosquito species. All searches were performed as decoy searches; a minimum score of 30 for at least one peptide was required for proteins to be reported. All the protein sequences were compared against the nonredundant database for homology searching through a BLASTP search (http://www.ncbi.nlm.nih.gov/BLAST/Blast.cgi?PAGE=Proteins) and SwissProt. Along with that, protein sequences were also searched against data of* An. stephensi* peptides present in VectorBase (https://www.vectorbase.org/organisms/anopheles-stephensi/). All identifications were manually validated and the proteins were selected and validated on basis of MOWSE scores, peptides matches, and % sequence coverage.

*OMSSA Server*. In order to evaluate other novel proteins which could not be detected with MASCOT, we have also used another search tool, that is, the open mass spectrometry search algorithm (ftp://ftp.ncbi.nih.gov/pub/lewisg/omssa/CURRENT/), under which probability score was used to determine specificity [13]. Here more spectra were matched as compared to other algorithms. Searches were made against* Anopheles gambiae* present in the taxonomy list. Almost all the parameters were kept the same for OMSSA as used earlier for MASCOT. All searches were conducted by using database SwissProt and NCBInr like MASCOT and only those peptides were reported as significant for which *E* value was statistically significant (*E* < 0.05).

## 3. Results and Discussion

Role of salivary glands and their proteins is important in the mosquito because parasites mature to form infectious sporozoites in salivary glands. Various active protein molecules must be annotated/expressed in salivary glands of mosquito which may help in food ingestion and digestion and facilitate blood feeding, immune defenses, and haemostasis [14]. In earlier studies various genes and their derived proteins have been studied in salivary glands of sugar fed* An. stephensi* by transcriptomics [3, 10]. Transcriptomics studies also identified transcripts and genes that may or may not be expressed at the protein level as some may be transcribed as nonfunctional sequences resembling functional genes. Proteomics studies however identify proteins directly and the corresponding genomic sequences can be designated as a protein-coding region. No attempts, however, have been made to study the detailed proteome of* An. stephensi* salivary glands for functional identification of such proteins.

Mass-spectrometry-based proteomics is now a powerful and reliable method that allows characterization of protein assemblies, and when this is combined with molecular, cellular, and bioinformatics techniques it provides a framework for translating complex molecules into simple molecules for in-depth analysis of expressed proteomes [15, 16]. Therefore, the goal of this study was to identify total salivary gland proteins of* An. stephensi* expressed by proteome analysis coupled with LC/MS/MS as an initial step towards the cataloging of the hundreds of proteins and peptides in the salivary proteome for future use in blood feeding experiments. The peak list/spectra obtained after LC/MS/MS were analyzed by both MASCOT (Matrix Science) and OMSSA algorithm and matched against databases of* Anopheles, Aedes, *and* Culex* mosquito species.

### 3.1. Mass Spectrometry-Based In-Gel Digested Sample Analysis

#### 3.1.1. MASCOT Algorithm

Availability of genome sequence for mosquito* An. gambiae* has led us to large-scale EST projects to identify potential genes and transcriptomes expressed in different mosquito tissues following blood meal. These EST projects are no doubt descriptive in nature and generate hypothesis on the evolution and function of genes [9]. Still that kind of analysis may identify abundant transcripts which might not be expressed at the protein level. However, if we directly identify and characterize a protein, the corresponding genomic transcript can be automatically designated as a protein-coding region.

**Figure 1:**
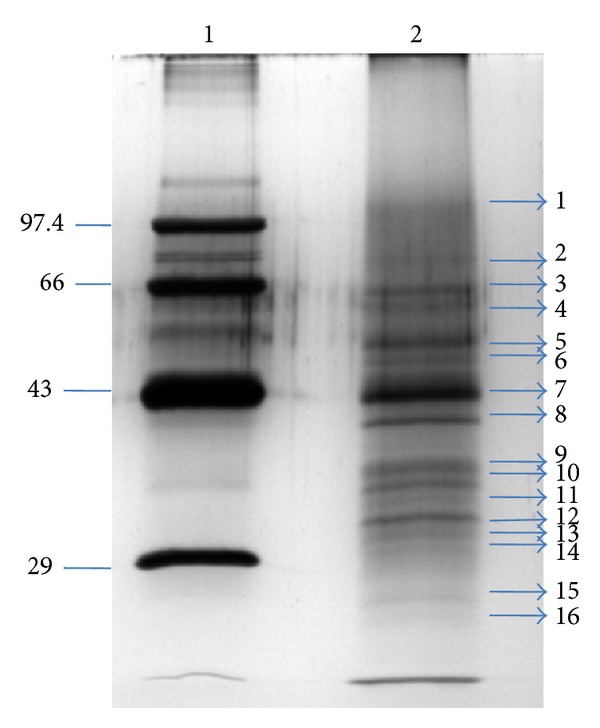
Salivary gland protein profiling of* An. stephensi.* Silver stained SDS-PAGE gel of the salivary gland extract is shown (lane 2). Protein markers with range 14 to 100 kDa are shown in lane 1.

In the present study, we employed a MS-based approach to categorize different putative functional proteins of salivary glands of an urban malaria vector* An. stephensi* of India. Total proteins of the salivary glands homogenate were first analyzed by in-gel approach on 10% SDS-PAGE. 16 gel bands of salivary gland homogenate sample were visualized after silver staining (Figure 1). In-gel digested peptides of* An. stephensi* salivary gland were then analyzed by LC/MS/MS. 36 known proteins and 12 novel proteins were identified by MASCOT algorithm. Some known salivary proteins and novel proteins and their details like molecular weight, peptides number, calculated pI, sequence coverage, and domain information are depicted in tables (Tables 1 and 2). Other known proteins are presented in supporting information (in Supplementary Material available online at http://dx.doi.org/10.1155/2014/686319).

Different proteins are also assigned according to gel bands. In Table 1 (including additional Table 1) 3 proteins are identified from band 2, 3 proteins from band 3, 1 protein from band 4, 2 proteins from band 5, 3 proteins from band 6, 4 proteins from band 7, 3 proteins from band 8, 1 protein from band 11, 1 protein from band 13, 4 proteins from band 14, 4 proteins from band 15, and 4 proteins from band 16. Same as in Table 2, 1 protein is identified from band 1, 1 protein from band 2, 1 protein from band 4, 1 protein from band 5, 1 protein from band 7, 2 proteins from band 13, 1 protein from band 14, 1 protein from band 15, and 4 proteins from band 16. These proteins with band number are given in respective tables (Tables 1 and 2).

Among all identified proteins by LC/MS/MS, further conserved domains were searched by using NCBI domain programs (www.ncbi.nlm.nih.gov/Structure/cdd/wrpsb.cgi) [17], Interproscan analysis and also predicted by SMART programme (http://smart.embl-heidelberg.de/) [18]. Signal peptides were also identified at the N-terminus of all identified proteins with the help of SignalP 4.1 (http://www.cbs.dtu.dk/services/SignalP/) which shows the indication of secretion [19].

**Figure 2:**
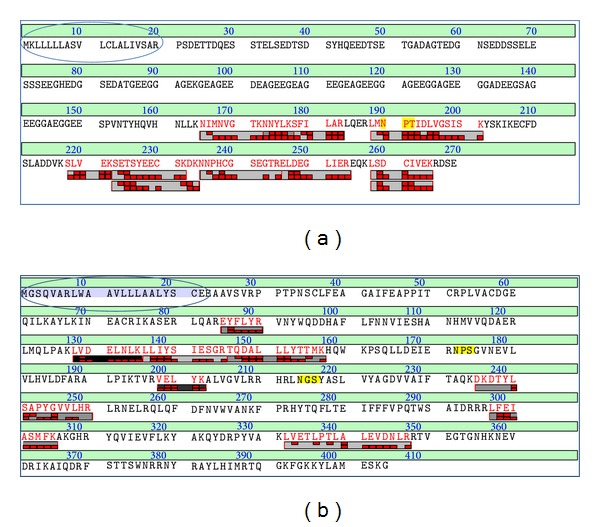
Annotated sequences of salivary protein precursors from* An. stephensi*. (a) GE rich salivary gland protein. The first 19 residues belong to the signal peptide of the precursor. (b) SG1D protein precursor. The first 24 residues belong to the signal peptide of the precursor.

Among the known proteins, GE rich salivary gland protein was found with 56% sequence similarity with the highest score (609) and a total of 8 peptide matches. Further signal peptide for GE rich salivary gland protein was identified at amino acid positions 1 to 19 which depicts a secreted protein (Figure 2(a)). Next to that D7 protein of score 587 with 14 peptide matches and significant threshold was identified with 34% sequence similarity. Others are like SG1D salivary protein precursor with 10 peptide matches (23%) and G1 family long salivary protein 3 (22% sequence similarity).

A sort of salivary gland proteins termed as SG1 family [20, 21] was identified with 11 to 23% sequence coverage. Signal peptide was identified among SG1 family. The position of signal peptide for SG1D salivary protein was identified at 1–24 (Figure 2(b)) and similarly for putative salivary protein SG1C 1 to 22 and putative salivary protein SG1A 1 to 26. Even earlier transcriptomics studies in* An. stephensi* also revealed about 9 cDNA clusters similarities to* An. gambiae* SG1 proteins family [3]. Valenzuela et al. also depicted five full-length sequences in* An. stephensi* that were related to different clusters of SG1 family [3] and a similar protein was identified in proteomic studies by LC/MS/MS analysis (SG1A, SG1B, SG1C, and SG1D) (Table 1). Other groups of proteins were identified which have secreted function like salivary apyrase (signal peptide at 1 to 27 position) and salivary antigen-5 related protein (signal peptide at 1 to 21 amino acid position).

13 novel hypothetical proteins were identified by MASCOT analysis that has features similar to proteins in other mosquito species like* An. gambiae*,* Aedes aegypti*,* Anopheles funestus*, and* Culex quinquefasciatus* (Table 2). One novel hypothetical protein identified was found similar to* Aedes aegypti* FOF1-type ATP synthase beta subunit with 16 peptide matches and 22% sequence similarity. Another protein identified was similar to Histone H4 of* Culex quinquefasciatus* (21%). One novel protein was found to be similar to tetraspanin of* An. gambiae* (signal peptide at position 1 to 33). This protein was well studied in* Drosophila* which is a conserved membrane protein and it was known to be involved in intracellular signaling and cellular motility [22].

#### 3.1.2. OMSSA Algorithm

All 16 digested samples were also analyzed by OMSSA algorithm after MS/MS analysis (ftp://ftp.ncbi.nih.gov/pub/lewisg/omssa/CURRENT/) by matching sequences of* An. gambiae* present in the database. We have identified and chosen only significant novel proteins matches on basis of *E* value. A total of 111 novel proteins from both SwissProt and NCBI nonredundant protein entries were identified by OMSSA algorithm. Information like molecular weight, % sequence coverage, and domain information of some putative functional significant proteins is depicted in Table 3. Other novel proteins are presented in supporting information. Many proteins were identified similar to gambicin (38%), glutaredoxin protein (34% sequence similarity), peroxidase 1 (23%), unknown protein (34%), CLIPB7 (31%), defender against apoptosis (28%), defensin (23%), peptidoglycan recognition protein 3 (22%), and so forth.

During LC/MS/MS analysis, one of the peptides eluted with an amino acid sequence NWATSGETVDECLEEMAGSACEQAYFFTRCVMTR was matched to putative odorant-binding protein OBPjj9 (*Anopheles gambiae*) that was analyzed by both b and y type ions. Signal peptide of this protein was identified at position 1 to 29 and 20% similarity was found. Many other proteins were also identified that have secreted function like defensin (signal peptide at position 1 to 25) and lysozyme c6 and protein O-fucosyltransferase 1 (signal peptide position for both was at 1 to 17).

**Figure 3:**
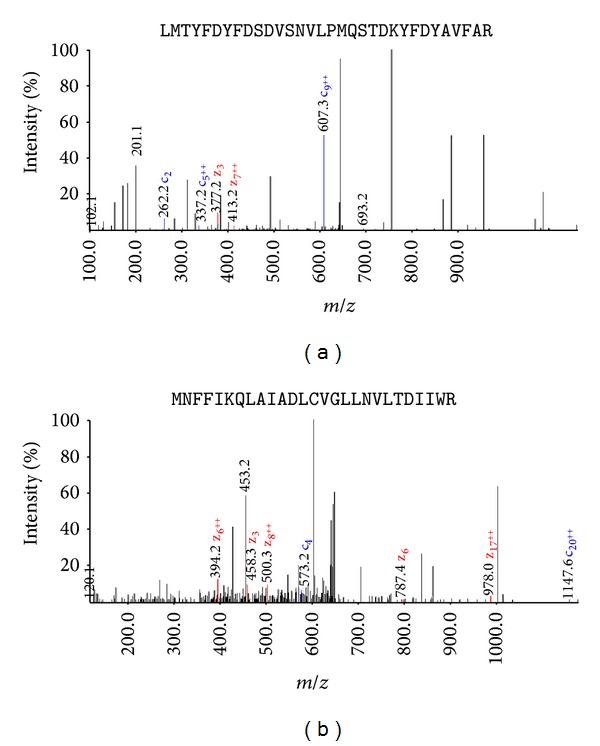
Peak spectrum analyzed by LC/MS/MS based on* m*/*z* values. (a) Peptide sequence (LMTYFDYFDSDVSNVLPMQSTDKYFDYAVFAR) with peak at* m*/*z* 470 in* An. stephensi* corresponds to novel protein (hexamerin) of* An. gambiae.* (b) Peptide sequence (MNFFIKQLAIADLCVGLLNVLTDIIWR) with peak at* m*/*z* 638 in* An. stephensi* corresponds to novel protein (G protein coupled receptor protein) of* An. gambiae*.

Another peptide with amino acid sequence LMTYFDYFDSDVSNVLPMQSTDKYFDYAVFAR was identified, that is, hexamerin, with signal peptide at position 1 to 18 (Figure 3(a)). A peptide sequence MNFFIKQLAIADLCVGLLNVLTDIIWR was identified similar to protein designated as G-protein coupled receptor in* An. gambiae* with peak spectrum at* m*/*z* 638 (Figure 3(b)).

### 3.2. Functional Significance of Identified Salivary Proteins

A total of 36 known proteins and 123 novel proteins were identified from both MASCOT and OMSSA algorithm. Putative functional annotation according to both biological approach and cellular approach was prepared among the identified proteins. These were identified by GO analysis (http://www.geneontology.org/).

**Figure 4:**
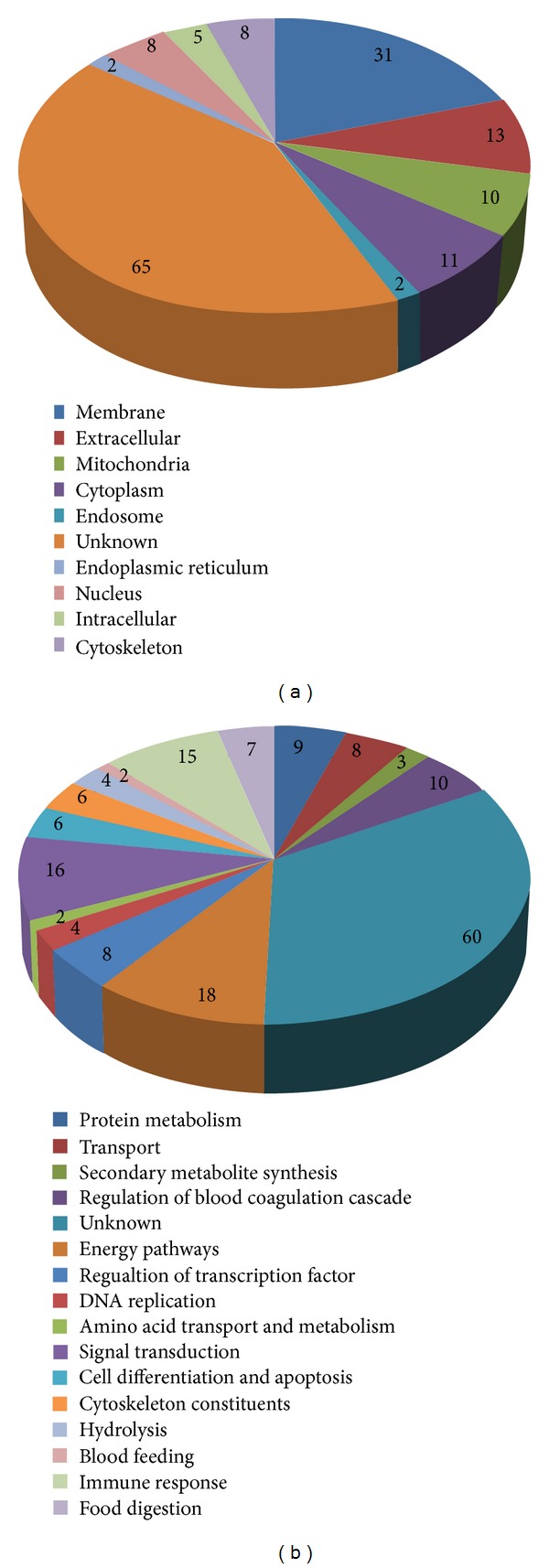
Depiction of identified salivary proteins of* An. stephensi* using gene ontology tool. (a) Intracellular localization of proteins identified by Q-TOF-MS/MS. (b) Functional (putative) classification of identified known and novel proteins. The number of total identified proteins is marked.

Subcellular location of each identified protein was assigned. We found most of the proteins localized in plasma membrane (31), extracellular (13), cytoplasm (11), mitochondria (10), nucleus (8), intracellular (5), cytoskeleton (8), and so forth. We are unable to find location of a large number of proteins that were assigned under unknown category (65) (novel or known) (Figure 4(a)).

On the basis of biological approach, the majority of proteins were scrutinized marked for their role in signal transduction, metabolism, cytoskeleton protein, transcription and translational regulation, energy pathways, regulation of blood coagulation cascade and intracellular trafficking and transport, stress response, and so forth (Figure 4(b)). 16 proteins fell within categories of signal transduction, 18 were categorized in electron carrier pathways, and so forth. Various housekeeping proteins were identified which act as cytoskeletal proteins like actin, myosin, tropomyosin, AGAP001799, and AGAP010147 playing a vital role in salivary gland. Proteins marked for chemosensory role were also found such as odorant-binding protein (OBP 52, OBPjj9, and OBP5470) and one hypothetical protein that has insect pheromone binding domain. Among D7 proteins (ancestral one, which was known to be originated from OBP proteins family), 6-cysteine and 10-cysteine residues are conserved and due to characteristic fold structure, they are distantly linked to OBP protein family [23, 24]. Though their functions are varied like OBP role as an odorant carrier to the olfactory receptors and the function of D7 proteins has been projected to facilitate blood feeding by inhibiting hemostasis [25–27].

Various proteins that play an important role in immune responses were identified such as defensins, fibrinogen binding proteins (FBN9), majority of serine proteases, CLIPB (CLIPB 14, CLIPB 15, CLIPB 7, and CLIPB 13), serine protease 14, immune factor (rel homology domain), and lysozyme c6. Such proteins may also be responsible for reduction in microbial load in ingested blood. Among them defensin protein in* An. stephensi* was found to be 23% similar to* An. gambiae* protein. After analyzing with SMART programme, interestingly we also found Knot1 domain which represents the antimicrobial peptides and has a role in defensive mechanism. In the salivary gland lysozyme c6 may help to check the bacterial growth in sugar meals of mosquitoes [28]. Various proteins involved in oxidoreductive process or stress response were also recognized like ND4L gene product, cytochrome 450, glutathione S-transferases 3–8, glutathione S-transferase E1, and glutathione S-transferase E4. Among stress proteins heat shock protein (hsp DNA J) was the one that mainly helps in providing defense against various external stresses [29]. Several proteins were also identified by proteomic studies that were not described earlier by transcriptomics study in* Anopheles stephensi* such as proteins involved in signal transduction as STAT protein, SCRBQ2 protein, Anlar, STAT 1, calpain, TEP 2 protein, and others involved in transport such as tryptophan transporter, nicotinic acetylcholine receptor subunit *β*1, and ACP receptor putative cation proton antiporter.

Long lists of enzymes were also identified that function as vasodilators, that is, peroxidases [30]. Three peroxidases enzymes, that is, peroxidase 1, peroxidase 12, and peroxidase 15, with 23–27% sequence similarity were identified. Even transcriptomics study also depicted 2 clusters of 12 sequences predict 80% sequences identical to* An. gambiae* [3]. Another enzyme which fell into antihemostatic category was salivary apyrases enzyme. The genes for these vasodilators or antihemostatic enzymes are expressed in the medial lobe and distal-lateral lobes of salivary gland [31–33]. This enzyme in insects is known to inhibit platelet aggregation by destroying the ATP and ADP released by activated platelets [31]. Transcriptomic studies of apyrase in* An. stephensi* also showed identity with salivary apyrase protein.

Among different tables some proteins with sequence coverage below 5% were identified which were actually not native proteins; in fact they are degraded product of the putative proteins.

### 3.3. Network Analysis of Known and Predicted Protein Interactions

**Figure 5:**
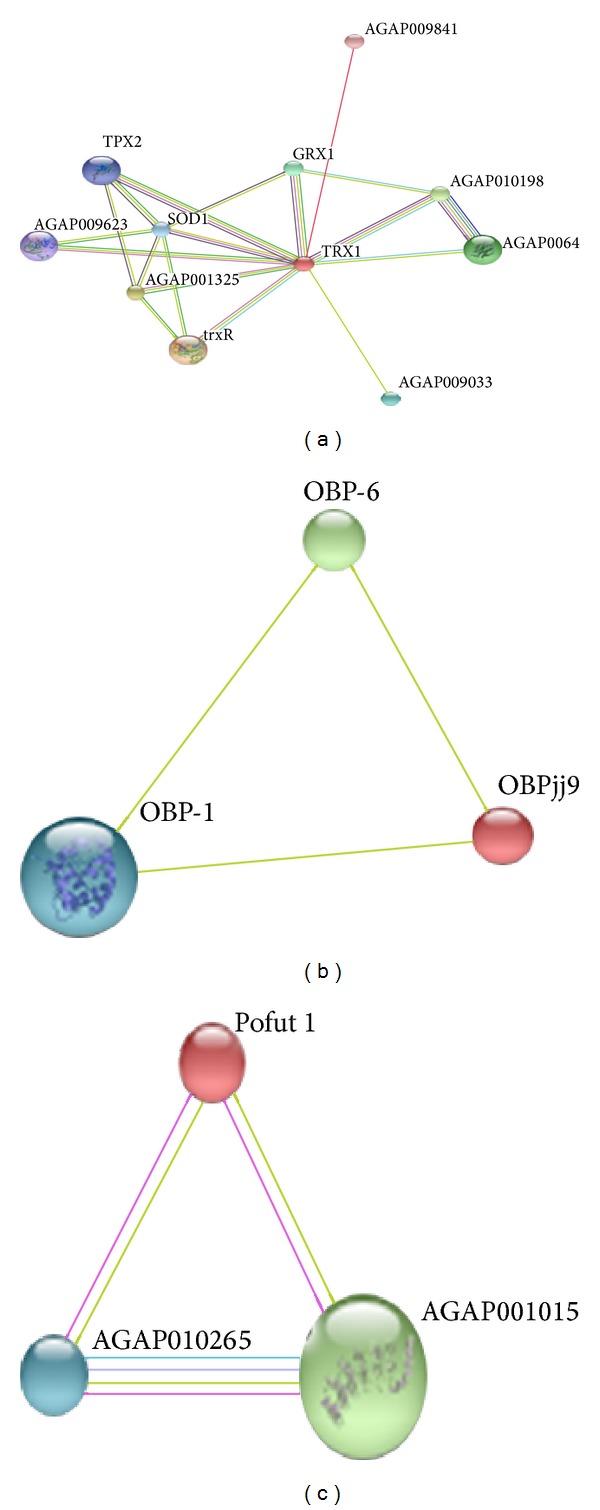
STRING network of protein-protein interactions identified by OMSSA algorithm. (a) Protein Q9NGZ1_ANOGA is shown as TRX1 (thioredoxin 1) in the network showing functional interactions with other proteins. (b) Protein Q7QCC4_ANOGA is shown as OBPjj9 (odorant-binding protein). (c) Protein Q7QHS7_ANOGA is shown as pofut1 (O-fucosyltransferase 1 protein). Different line colors represent the types of evidence for the association. Green color depicts neighborhood; red color: gene fusion; pink color: experiments; light green color: text mining; blue color: cooccurrence; dark blue color: coexpression; purple color: homology. Circle nodes indicated different proteins.

We also presented the STRING network of some known/novel protein-protein interactions as an evidence view by using String 9.0 (Search Tool for the Retrieval of Interacting Genes/Proteins) database of physical and functional interactions (http://string-db.org/) [34]. String 9.0 helps in predicting functional partners with a database of known and predicted protein interactions. Such protein-protein interactions are further important for signaling pathways studies and modeling studies of complex proteins. Evidence view of novel thioredoxin protein (21% similarity) identified by OMSSA algorithm was shown as interactions with other proteins, that is, glutaredoxin, superoxide dismutases, thioredoxin reductase, thioredoxin dependent peroxidases, and so forth (Figure 5(a)), which may predict further its significance in signaling pathways. Evidence view of odorant-binding protein showed interaction with other odorant-binding proteins (OBP6, OBP1) and their precursors and protein O-fucosyltransferase 1 with other* An. gambiae* proteins (Figures 5(b) and 5(c)).

## 4. Conclusions

Mass spectrometric based proteomics techniques coupled with high throughput bioinformatic analysis are a powerful platform to understand comprehensive biology and interaction of functional proteins. Salivary gland proteins of the* Anopheles* mosquitoes are believed to be important in the development of the plasmodium as these molecules are involved in the antihemostatic activity, which may assist during the blood feeding process and play a critical role in the transmission of malaria parasite. Our idea was to analyze the putative functional role of the previously known and other novel salivary proteins that may be essential for parasite development in the mosquito directly or indirectly. Here, we report our initial studies using proteomic approaches to characterize the salivary gland proteomic repertoire of an urban malaria vector* An. stephensi* and its identification by searching protein sequence databases. For that, two different algorithms were used to identify the proteins or peptides from databases (NCBI nr, SwissProt), namely, MASCOT and OMSSA, and a total of 36 known proteins and 123 novel proteins were analyzed. These identified proteins were analyzed functionally (molecular and biological) by using bioinformatics software so that such salivary proteins can be further employed to understand the concept of feeding, insecticide resistance mechanisms, signal transduction, immunological properties, and various aspects of vector-parasite host interactions.

Such proteins may be used for development of novel antimalarial control strategies for improving innate protection against malaria and help to elucidate the various aspects of salivary gland-malaria parasite interactions.

## Supplementary Material

Additional Table 1: Long list of known proteins identified by using in-gel digestion strategy using MASCOT algorithm.
Additional Table 2: Long list of novel proteins identified by using in-gel digestion strategy using OMSSA algorithm.
Additional table 2 is the supporting information mentioned in Results & discussion section 3.1.2 OMSSA algorithm (11^th^ line).

## Figures and Tables

**Table 1:** A catalogue of known proteins identified by using in-gel digestion strategy and LC/MS/MS using MASCOT algorithm.

S. number	Accession number/vector base accession number	Protein	Band number	Mol. weight	Peptides	Calculated pI	Sequence coverage	Domain/function
1	gi: 37201975	GE rich salivary protein	16	15214	8	5.15	56%	No conserved domain
2	gi: 15718081	D7 protein	11	36396	14	8.79	34%	Protein binding/glycoprotein
3	gi: 29501536	SG1D salivary protein precursor	7	46811	10	9.38	23%	No conserved domain
4	ASTM013042-PA	G1 family long form salivary protein 3	7	45829	9	6.85	22%	No conserved domain
5	gi: 27372941	Putative salivary protein SG1C	7	44292	8	6.73	16%	No conserved domain
6	gi: 27372911	Salivary apyrase	4	64248	8	6.77	12%	No conserved domain
7	ASTM006960-PA	Alpha amylase	3	67923	2	5.27	4%	Alpha amylase domain/catalytic activity
8	ASTM007102-PA	Salivary peroxidase	3	67504	6	8.75	10%	Heme binding/peroxidase activity
9	gi: 27372939	Putative salivary protein SG1A	15	19725	2	4.94	11%	Nucleotide transport and metabolism
10	gi: 27372929	Putative salivary protein SG1B	6	48120	2	—	4%	No conserved domain
11	gi: 29501376	Short D7-4 salivary protein precursor	15	18412	1	—	7%	No conserved domain
12	gi: 27372895	Salivary antigen-5 related protein	14	28974	2	9.05	8%	CTD-interacting domain (polypeptide binding)
13	gi: 29501528	TRIO salivary gland protein precursor	7	44013	3	7.01	3%	SCP-like extracellular protein domain

**Table 2:** A catalogue of novel proteins identified by using in-gel digestion strategy and LC/MS/MS using MASCOT algorithm.

S. number	Accession number	Protein	Band number	Mol. weight	Peptides	Calculated pI	Sequence coverage	Domain/function
1	gi: 94468834	FOF1-type ATP synthase beta subunit (similar to *Aedes aegypti*)	5	53937	16	5.03	22%	Nucleotide-binding domain
2	gi: 170059752	Histone H4 (similar to *Culex quinquefasciatus*)	16	11374	2	11.36	21%	Nucleosome assembly
3	gi: 118784826	AGAP005078-PA (similar to *Anopheles gambiae* str. PEST)	16	13032	2	11.16	13%	No conserved domain
4	gi: 347963754	AGAP000403-PA (similar to *Anopheles gambiae* str. PEST)	15	19831	1	10.37	6%	Nucleotide binding
5	gi: 356578763	Copper/zinc superoxide dismutase 3B (similar to *An. aquasalis*)	16	15646	1	5.94	6%	Ion binding
6	gi: 118782571	AGAP002575-PA (similar to *Anopheles gambiae* str. PEST)	14	27619	1	5.03	3%	Leucine rich repeats
7	gi: 129716442	Rps7 (fragment) OS similar to *Anopheles funestus *	16	15374	1	9.85	3%	Translation
8	*gi*: *122116875 *	Molybdenum cofactor sulfurase 2 (similar to *Aedes aegypti) *	2	85615	2	6.76	3%	Pyridoxal phosphate- (PLP-) dependent enzymes
9	gi: 158285167	AGAP007706-PA (kinesin-like protein)	1	99191	3	9.2	2%	ATP activity
10	gi: 158299522	Tetraspanin protein (similar to *Anopheles gambiae* str. PEST)	13	29075	1	8.96	2%	No conserved domain
11	gi: 58391886	AGAP009833-PA (similar to *Anopheles gambiae* str. PEST)	13	30740	2	8.64	2%	Porin
12	gi: 170037149	Apoptosis inhibitor (similar to *Culex quinquefasciatus*)	4	61233	2	6.46	1%	No conserved domain

**Table 3:** A catalogue of novel proteins identified by using in-gel digestion strategy and LC/MS/MS using OMSSA algorithm.

S. number	Accession	Features	MW	% Seq coverage	*E* value	Domain
1	gi: 224037899	Gambicin (similar to *Anopheles gambiae*)	3373.62	38%	0.02	No conserved domain
2	gi: 126680249	Unknown (similar to *Anopheles gambiae*)	5546.78	34%	0.02	No conserved domain
3	gi: 53771806	Glutaredoxin (similar to *Anopheles gambiae*)	1932.89	34%	0.005	GRX domain
4	gi: 126680357	Unknown (similar to *Anopheles gambiae*)	3266.66	33%	0.045	No conserved domain
5	gi: 187440102	CLIPB7 protein (similar to *Anopheles gambiae*)	4640.28	31%	0.01	Clip domain
6	gi: 31281916	Xanthine dehydrogenase (similar to *Anopheles gambiae*)	1641.8	29%	0.05	Fe-S cluster binding domain
7	gi: 37576232	Defender against programmed cell death (similar to *Anopheles gambiae*)	3491.73	28%	0.05	Integral membrane protein
8	gi: 5834921	ND4L gene product (mitochondrion) (similar to *Anopheles gambiae*)	3456.69	28%	0.01	Oxidoreductases
9	gi: 54124659	Peroxidase 12 (similar to *Anopheles gambiae*)	3793.88	27%	0.009	Peroxidase domain
10	gi: 187440702	GNBPB1 protein (similar to *Anopheles gambiae*)	3776.06	26%	0.02	No conserved domain
11	gi: 87080401	Putative TIL domain polypeptide (similar to *Anopheles gambiae*)	3276.31	25%	0.03	Trypsin inhibitor-like cysteine rich domain
12	gi: 3139135	Defensin (similar to *Anopheles gambiae*)	2263	23%	0.004	Defensin superfamily
13	gi: 54124633	Peroxidase 1 (similar to *Anopheles gambiae*)	1978.95	23%	0.02	Animal heme peroxidases
14	gi: 281186343	Peptidoglycan recognition protein 3 short class (similar to *Anopheles gambiae*)	4460.29	22%	0.002	Pattern recognition receptor
15	gi: 18139597	Cytochrome P450 CYP4C28 (similar to *Anopheles gambiae*)	3816.62	22%	0.01	cypX domain
16	gi: 187340440	PGRPS1 protein (similar to *Anopheles gambiae*)	1596.76	21%	0.001	Peptidoglycan recognition proteins (PGRPs)
17	gi: 7716428	Thioredoxin 1 (similar to *Anopheles gambiae*)	2425.01	21%	0.02	TRX domain
18	gi: 37677930	agCP14332 (similar to *Anopheles gambiae*)	2083.04	21%	0.001	No conserved domain
19	gi: 158452713	Caspase short class, partial (similar to *Anopheles gambiae*)	1869.74	21%	0.038	No conserved domain
20	gi: 48994192	Putative odorant-binding protein OBPjj9 (similar to *Anopheles gambiae*)	3964.71	20%	0.02	Olfactory receptor, OBP
21	gi: 6635469	Immune-responsive trypsin-like serine protease-related protein ISPR10 (similar to *Anopheles gambiae*)	2705.24	20%	0.004	No conserved domain
22	gi: 187441150	SCRB2 protein (similar to *Anopheles gambiae*)	2410.2	19%	0.05	Scavenger receptor
23	gi: 40019419	Odorant-binding protein OBP5470 (similar to *Anopheles gambiae*)	3655.7	18%	0.01	No conserved domain
24	gi: 28396160	Putative antennal carrier protein AP-1 (similar to *Anopheles gambiae*)	2793.37	18%	0.03	No conserved domain
25	gi: 310756184	AGAP005196 (similar to *Anopheles gambiae*)	2911.43	18%	0.02	Tryp_SPc domain
26	gi: 13509402	Hypothetical protein (similar to *Anopheles gambiae*)	2117	18%	0.02	No conserved domain
27	gi: 37703114	Odorant receptor 1 (similar to *Anopheles gambiae*)	4085.1	17%	0.002	Transmembrane receptor
28	gi: 187441612	TEP2 protein (similar to *Anopheles gambiae*)	3276.31	16%	0.03	Terpene cyclases domain
29	gi: 2564570	NADH dehydrogenase subunit 5 (similar to *Anopheles gambiae*)	4157.12	16%	0.003	Ubiquitin/PQ complex
30	gi: 187440738	CLIPB13 protein (similar to *Anopheles gambiae*)	2515.41	14%	0.05	Trypsin like serine protease
31	gi: 311985	ANG12 precursor (similar to *Anopheles gambiae*)	3171.52	14%	0.06	Insect allergen related repeat
32	gi: 187441890	SCRBQ2 protein (similar to *Anopheles gambiae*)	2356.32	14%	0.001	CD36 family
33	gi: 187444412	FBN9 protein (similar to *Anopheles gambiae*)	2425.3	14%	0.01	No conserved domain
34	gi: 1495237	GSTD2 protein (similar to *Anopheles gambiae*)	3391.77	14%	0.005	*α* helical domain GS transferases
35	gi: 1369924	Immune factor (similar to *Anopheles gambiae*)	2675.3	13%	0.07	Rel homology domain
36	gi: 19071278	Odorant-binding protein (similar to *Anopheles gambiae*)	2129.03	13%	0.06	Olfactory receptor
37	gi: 117957967	Beta carbonic anhydrase (similar to *Anopheles gambiae*)	3784.98	13%	0.008	Carbonic anhydrase domain
38	gi: 33355867	Odorant-binding protein AgamOBP52 (similar to *Anopheles gambiae*)	2645.31	13%	0.002	No conserved domain
39	gi: 12007372	Glutathione S-transferase E1 (similar to* Anopheles gambiae*)	2884.43	12%	0.001	GSTC Delta epsilon
40	gi: 1245442	Putative arylphorin precursor, partial (similar to *Anopheles gambiae*)	2681.36	11%	0.02	Copper containing protein
41	gi: 240270034	Serpin 7 inhibitory serine protease inhibitor (similar to *Anopheles gambiae*)	3358.47	11%	0.002	Proteinase inhibitors
42	gi: 157042594	suppressor of cytokine signaling 5 (similar to *Anopheles gambiae*)	2507.2	11%	0.03	SH2 domains
43	gi: 169260669	Vasa (similar to *Anopheles gambiae*)	1458.78	11%	0.03	Helicase C terminal domain
44	gi: 71841593	pk-1 receptor (similar to *Anopheles gambiae*)	4041.93	11%	0.001	G protein coupled receptor
45	gi: 853701	Serine proteinase (similar to *Anopheles gambiae*)	2735.34	11%	0.02	Trypsin-like serine protease
46	gi: 1256440	Hexamerin (similar to *Anopheles gambiae*)	3847.48	5%	0.03	Hemocyanin Ig-like domain

*OMSSA: open mass spectrometry search algorithm.
