# Supplementary material for: Mass Spectrometry Based Proteomic Analysis of Salivary Glands of Urban Malaria Vector Anopheles stephensi
Source: Biomed Res Int. 2014 Jul 14;2014:686319. doi: 10.1155/2014/686319 (PMC4122192; doi:10.1155/2014/686319)
Supplement: Supplementary file 1 — Additional Table 1: Long list of known proteins identified by using in-gel digestion strategy using MASCOT algorithm. Additional Table 2: Long list of novel proteins identified by using in-gel digestion strategy using OMSSA algorithm. Additional table 2 is the supporting information mentioned in Results & discussion section 3.1.2 OMSSA algorithm (11th line). [file 686319.f1.pdf]

Supplementary Table 1: A catalogue of known proteins identified by using in-gel digestion strategy and LC/MS/MS using MASCOT algorithm

S.no	Accession number/vector base Accession number	Protein	Mol. Weight	Peptides	Calculated pI	Sequence coverage	Domain/Function
1	ASTM005454-PA	Tropomyosin 2	30832	5	4.82	17%	No conserved domain
2	ASTM006933-PA	Myosin heavy chain	135972	5	5.2	4%	Motor activity
3	ASTM007096-PA	sulfate transporter protein	88306	2	9.27	2%	Transport
4	ASTM014183-PA	Hypothetical protein/ATP synthase alpha subunit	80465	2	9.81	2%	ATPase enzyme
5	GI: 339649279	Glutathione S-Transferase E4	25076	1	6.53	5%	No conserved domain
6	ASTM000527-PA	Hypothetical protein	66553	1	8.09	1%	uncharacterized
7	ASTM017293-PA	Porin (voltage dependent anion selective channel)	42907	1	9.12	1%	Anion transport
8	ASTM005798-PA	elongation factor1 alpha 1	59939	1	8.71	1%	GTP binding
9	ASTM010693-PA	short chain dehydrogenase	27502	2	7	8%	oxidoreductase function
10	ASTM014983-PA	Histone H2A	13355	2	10.72	11%	nucleosome assembly/DNA binding
11	ASTM009772-PA	Actin	41852	2	5.36	7%	Actin protein HSP70/ATP binding
12	ASTM015472-PA	Hypothetical protein	21893	1	8.29	10%	Uncharacterized
13	ASTM005771-PA	Peptide N-glycanase	85828	2	8.77	1%	signal transduction
14	ASTM009670-PA	Prefoldin subunit	18939	1	5.66	4%	[Posttranslational modification, protein turnover, chaperones]

15	ASTM017244-PA	JmjC domain-containing histone demethylation protein	48571	1	9.07	2%	protein binding
16	ASTM000815-PA	Hypothetical protein	181818	2	8.47	1%	Signal transduction
17	ASTM001058-PA	Hypothetical protein (Rho effector protein)	15505	1	9.16	5%	Signal transduction
18	GI:373880220	Calreticulin	46514	2	4.37	2%	Unfolded protein binding
19	ASTM007078-PA	Axonemal dynein light chain	28779	1	5.5	2%	Motility
20	ASTM017761-PA	Type -2 keratin	56560	2	7	3%	Cell division and chromosome partitioning
21	ASTM012154-PA	Hypothetical protein	7046	1	6.9	11%	Uncharacterized
22	GI:380865625	BZIP1 OS	41596	3	8.85	4%	DNA binding
23	GI:66863200	Putative reverse transcriptase (Fragment)	13461	1	9.93	4%	RNA-directed DNA polymerase activity

Supplementary Table 2: A catalogue of novel proteins identified by using in-gel digestion strategy and LC/MS/MS using OMSSA algorithm

S.no	Accession	Features	MW	% Seq Coverage	E value	Domain
1	gil3617768	nitric oxide synthase [similar *to Anopheles gambiae*]	2321.1	14%	0.04	Ferrous reductase transfer
2	gil46948820	maverick [similar to *Anopheles gambiae*]	2205.03	14%	0.05	TGFβlike domain
3	gil159032230	zinc finger protein 294 [similar to *Anopheles gambiae*]	3058.42	14%	0.03	No conserved domain
4	gil148616061	transposase [similar to *Anopheles gambiae*]	2471.09	14%	0.002	Zinc finger domain
5	gil90654898	short neuropeptide F prepropeptide [similar to *Anopheles gambiae*]	3491.73	13%	0.049	No conserved domain
6	gil3170241	TU37B2 [similar to *Anopheles gambiae*]	2886.33	13%	0.005	Integral membrane protein
7	gil15485212	calpain [similar to *Anopheles gambiae*]	2763.78	12%	0.01	Cysteine proteinases , calpain domain
8	gil145559280	CTLMA2 [similar to *Anopheles gambiae*]	1988.89	11%	0.04	Ctype lectin
9	gil4099803	Tc1-like transposase [similar to *Anopheles gambiae*]	3921.89	10%	0.02	Transposase domain
10	gil77415700	hypothetical protein [similar to *Anopheles gambiae*]	1939.92	10%	0.03	Insect pheromone binding family
11	gil37962892	ICHIT [similar to *Anopheles gambiae*]	3580.8	10%	0.055	Chitin binding domain
12	gil119393795	Rab5 [similar to *Anopheles gambiae*]	2223.13	10%	0.008	Rab realted GTPase family, nucleotide Po_4 bindind motif
13	gil37703102	FBN23 [similar to *Anopheles gambiae]*	3325.7	10%	4.94E-05	No conserved domain
14	gil40792589	superoxide dismutase 1 [similar to *Anopheles gambiae*]	2212.16	9%	0.04	Fe-Mn superoxide dismutase

15	gi|62528997	CAPA-like receptor [similar to *Anopheles gambiae]*	4301.27	9%	0.003	No conserved domain
16	gi|6635467	immune-responsive serine protease-related protein ISPR9 [similar to *Anopheles gambiae*]	1754.84	9%	0.05	No conserved domain
17	gi|18140727	twelve cysteine protein 1 [similar to *Anopheles gambiae*]	1622.22	9%	0.007	No conserved domain
18	gi|56067723	alanyl-tRNA synthetase [similar to *Anopheles gambiae*]	3093.48	9%	0.0007	tRNA synthetase
19	gi|1220128	vacuolar ATPase [similar to *Anopheles gambiae*]	1425.68	9%	0.04	F subunit
20	gi|187441860	CLIPB15 protein [similar to *Anopheles gambiae*]	1761.77	9%	0.03	Trypsin like serine proteases
21	gi|52429829	fruitless female-specific zinc-finger C isoform [similar to *Anopheles gambiae*]	5456.18	9%	0.02	No conserved domain
22	gi|224038197	serine protease 14 [similar to *Anopheles gambiae]*	3277.52	9%	0.02	Trypsin like serine proteases
23	gi|63259109	CYP325C2 [similar to *Anopheles gambiae*]	2376.09	8%	0.047	Cyp x domain
24	gi|38196173	SRPN9 [similar to *Anopheles gambiae*]	2298.15	8%	0.0005	Proteinase inhibitors
25	gi|19577378	putative translation initiation factor [similar to *Anopheles gambiae*]	3156.38	8%	0.034	Salvage domain
26	gi|28396156	putative antennal carrier protein TOL-1 [similar to *Anopheles gambiae*]	2636.35	8%	0.013	Juvenile hormone
27	gi|38708297	SP22D [similar to *Anopheles gambiae*]	2217.18	7%	0.01	Chitin binding domain
28	gi|2253393	serine protease 14D [similar to *Anopheles gambiae*]	2613.33	7%	0.04	Trypsin like serine protease
29	gi|56067837	heat shock protein DnaJ [similar to *Anopheles gambiae*]	1368.7	7%	0.04	Thioredox domain

30	gi|253970801	putative Ropn1l-like protein [similar to *Anopheles gambiae*]	4970.35	7%	0.04	No conserved domain
31	gi|47606680	G-protein coupled receptor 4 [similar to *Anopheles gambiae*]	3301.71	7%	0.03	transmembrane receptor
32	gi|2076747	engrailed [similar to *Anopheles gambiae*]	3411.61	6%	0.002	No conserved domain
33	gi|325699280	AGAP007032-PA [similar to *Anopheles gambiae*]	2639.15	6%	0.035	No conserved domain
34	gi|3128484	dopa decarboxylase isoform 2 [similar to *Anopheles gambiae*]	3316.63	6%	0.06	Pyridoxal $P0_4$ dependent
35	gi|327244539	APL1B [similar to *Anopheles gambiae*]	3546.66	6%	0.009	Leucine rich repeat
36	gi|14572580	arrestin [similar to *Anopheles gambiae*]	2245.27	6%	0.004	S antigen C like domain
37	gi|21541544	glutathione S-transferase 3-8 [similar to *Anopheles gambiae*]	1649.07	6%	0.01	GST domain
38	gi|160905682	ACP receptor [similar to *Anopheles gambiae*]	3301.71	6%	0.03	7 transmembrane receptor
39	gi|239819632	carbonic anhydrase 4 [similar to *Anopheles gambiae*]	2457.2	6%	0.016	No conserved domain
40	gi|577351	putative nucleic acid binding protein [similar to *Anopheles gambiae*]	2433.2	6%	0.01	No conserved domain
41	gi|187447804	ENSANGG00000009053 protein [similar to *Anopheles gambiae*]	1584.76	6%	0.001	No conserved domain
42	gi|19572383	putative glycerol kinase [similar to *Anopheles gambiae*]	2884.43	5%	0.01	Kinase,HSP70
43	gi|51873097	nicotinic acetylcholine receptor subunit beta 1 [similar to *Anopheles gambiae]*	3427.58	5%	0.002	Neurotransmitter type domain
44	gi|28569863	gag-like protein [similar to *Anopheles gambiae*]	2485.18	5%	0.04	No conserved domain

45	gi|17932668	Ag9 protein [similar to *Anopheles gambiae]*	1996.87	5%	0.01	apyrase
46	gi|270266159	plugin [similar to *Anopheles gambiae*]	3331.58	5%	0.024	No conserved domain
47	gi|284928365	CLIPB14 [similar to *Anopheles gambiae*]	2193.06	5%	0.001	Trypsin like serine protease
48	gi|118500886	gustatory receptor 22 [similar to *Anopheles gambiae*]	2735.31	5%	0.0001	No conserved domain
49	gi|225675537	signal transducer and activator of transcription [similar to *Anopheles gambiae*]	4579.86	5%	0.01	STAT protein, α domain
50	gi|75911308	protein O-fucosyltransferase 1 [similar to *Anopheles gambiae*]	2446.33	5%	0.001	GDP fucose protein
51	gi|62529001	pyrokinin-like receptor 2 [similar to *Anopheles gambiae*]	2723.4	4%	0.01	No conserved domain
52	gi|226711462	RecName: Full=Cytoplasmic tRNA 2-thiolation protein 2 (similar to *An. gambiae*)	2026.97	4%	0.01	No conserved domain
53	gi|145413657	LRIM1 [similar to *Anopheles gambiae*]	2111.07	4%	0.002	No conserved domain
54	gi|54124667	peroxidase 15 [similar to *Anopheles gambiae*]	1289.6	4%	0.002	Animal heme peroxidases
55	gi|156028184	AGAP002429-PA [similar to *Anopheles gambiae*]	2333.27	4%	0.0003	CypX
56	gi|3406737	S-adenosyl-L-homocysteine hydrolase [Anopheles gambiae]	2103.76	4%	0.004	Hydrolases domain
57	gi|222160938	putative cation proton antiporter [similar to *Anopheles gambiae*]	2309.05	4%	0.01	K^+ transport
58	gi|46948822	medea [similar to *Anopheles gambiae]*	3546.68	4%	0.02	MH_2 domain
59	gi|19577386	putative chitin binding protein [similar to *Anopheles gambiae]*	2310.17	4%	0.008	No conserved domain

60	gi|6759388	putative serine protease inhibitor [similar to *Anopheles gambiae*]	1502.76	3%	0.037	Chaperonins proteins
61	gi|62911114	lysozyme c-6 [similar to *Anopheles gambiae*]	2801.14	3%	0.009	C type lysozyme
62	gi|732550	Anlar [similar to *Anopheles gambiae]*	3760.79	3%	0.003	Tyrosine phosphatases
63	gi|46981770	tryptophan transporter [similar to *Anopheles gambiae*]	2361.14	3%	0.005	Sodium neurotransmitter
64	gi|332330728	chitinase 5-3 [similar to *Anopheles gambiae*]	1524.78	3%	0.012	GH18 type II glyco18 domain
65	gi|327244332	APL1A [similar to *Anopheles gambiae]*	1735.07	3%	0.005	No conserved domain

*OMSSA: Open mass spectrometry search algorithm
